# When Pain Outlasts Our Drugs: The Case for Sustained-Release Analgesia in Sheep

**DOI:** 10.3390/ani16030505

**Published:** 2026-02-05

**Authors:** Mahla Nateghi Baygi, Lee Narelle Metcalf, Benjamin Kimble, Sabrina Lomax

**Affiliations:** 1 School of Life and Environmental Sciences, Faculty of Science, The University of Sydney, Sydney, NSW 2006, Australia; mahla.nateghibaygi@sydney.edu.au; 2 Sydney School of Veterinary Science, Faculty of Science, The University of Sydney, Sydney, NSW 2006, Australia; lee.metcalf@sydney.edu.au (L.N.M.); benjamin.kimble@sydney.edu.au (B.K.); 3 The University of Sydney Institute of Agriculture, The University of Sydney, Sydney, NSW 2006, Australia

**Keywords:** sheep welfare, pain relief, livestock husbandry, NSAIDs, pharmacokinetics

## Abstract

Pain associated with routine husbandry procedures in sheep can persist for several days or even weeks, yet current analgesic options, such as the non-steroidal anti-inflammatory drug (NSAID) meloxicam, typically provide only 24–36 h of analgesia. This mismatch between pain duration and analgesic coverage represents a fundamental limitation of current pain-management strategies in sheep. Sustained-release (SR) formulations are emerging as a promising approach to deliver longer-lasting pain relief from a single dose, reducing the need for repeated handling, and improving both animal welfare and farm efficiency. Emerging evidence highlights both the promise and limitations of different strategies to extend therapeutic coverage beyond 72 h. While preliminary results are encouraging, challenges remain in achieving consistent pharmacokinetic profiles and optimal peak concentrations. Advancing SR meloxicam formulations could support widespread uptake of welfare-focused innovations in the sheep industry. Although not yet widely available, sustained-release meloxicam represent a promising step towards making routine husbandry procedures more humane and efficient.

## Simple Summary

Routine husbandry procedures in sheep can cause pain that lasts for several days or longer, but current pain relief options usually provide only short-term relief. Because sheep are rarely re-handled after routine procedures, pain relief strategies that require repeated dosing are poorly aligned with commercial sheep production systems. Sustained-release formulations of the pain-relieving drug meloxicam are being developed to deliver longer-lasting analgesia from a single treatment, reducing the need for repeated handling of animals. Recent studies suggest these formulations have the potential to improve animal welfare and on-farm efficiency, although further work is needed to ensure consistent and reliable pain relief. Overall, sustained-release meloxicam represents a promising approach to improving pain management during routine sheep husbandry procedures.

## 1. The Persistent Problem of Pain Management in Livestock

With the growing global demand for textiles and animal-derived foods, the sheep industry plays a crucial role in meeting agricultural needs both in Australia and worldwide [1]. To maintain productivity and prevent welfare-related conditions, millions of sheep undergo routine husbandry procedures such as castration, tail docking, and mulesing (the surgical removal of skin around the breech area) each year [2]. Although these interventions are intended to promote long-term welfare, they inherently cause tissue damage and inflammation, leading to both acute and prolonged pain. Studies show that pain caused by routine procedures can last from several days to many weeks, with reported durations ranging from 3 to 112 days in different studies [3]. Pain responses such as abnormal posture, reduced activity and elevated cortisol levels have been observed well beyond the initial procedure [4]. Many of these trials concluded while animals were still showing clear signs of pain, indicating that discomfort may persist even longer than documented [3]. Despite growing recognition of this sustained pain, routine husbandry remains a pressing animal welfare concern. In recent years, there has been an increase in public awareness and societal concern for farm animal welfare driving changes within the livestock sector. Consumers increasingly demand that production systems align with humane standards [2,5]. In response, governments have implemented regulatory measures, or guidelines and recommendations to ensure the responsible management of pain in livestock. As an example, the Australian Animal Welfare Standards and Guidelines for Sheep recommends that lambs older than six months must receive pain relief during tail docking and mulesing and strongly recommend remaining updated on recent and effective approved analgesics for all painful husbandry procedures [6]. Similarly, in the UK, the Animal Welfare Committee has advised that castration and tail docking are associated with significant acute and longer-term pain and welfare impacts in lambs, and recommends their use only where clearly justified, alongside effective pain mitigation and consideration of less invasive alternatives [7].

Despite this recent progress, substantial gaps persist in the consistent implementation of analgesia across livestock industries. In Australia, for example, while almost all producers who perform mulesing now administer topical pain relief, only a small proportion (around 8%) combine it with a systemic analgesic such as meloxicam to provide extended pain mitigation [8]. For other routine husbandry procedures, uptake remains much lower, with less than half using pain relief for castration or tail docking [8]. These findings suggest that barriers to wider adoption of analgesic use may reflect the additional handling time and costs associated with current analgesic approaches, alongside perceptions regarding necessity, highlighting the need for effective and practical strategies capable of providing sustained pain relief without increasing handling time or cost.

## 2. Out of Sight, Still in Pain

Pain following routine husbandry in sheep is frequently under-recognised because clinical and behavioural indicators are subtle and easily missed as livestock are mostly prey animals and habitually mask their pain [3]. Behavioural changes such as reduced activity, isolation from the flock, lowered head carriage, altered posture, or decreased feeding [9,10], make pain less likely to be recognised by under routine farm conditions. Animals are typically returned to paddocks and reunited with their mothers as soon as possible following husbandry procedures to minimise additional stress which means that animals are rarely monitored beyond the immediate post-procedural period. Without extended observation, delayed pain behaviours such as stiffness, reduced grazing, and social withdrawal can go unnoticed [3]. Lack of post-operative monitoring for these signs can reinforce assumptions of rapid recovery, perpetuating the perception that pain relief is unnecessary.

Studies across livestock species indicate that untreated pain leads to reduced feed intake, slows growth and increases sensitivity to future stimuli [3]. Early or repeated exposure to pain can induce long-term sensitisation of neural pathways, lowering the threshold for future pain responses [3]. Long-term pain-related behaviours and physiological changes can suppress growth and fertility, which would all impact the overall productivity of the farm and could therefore have economic consequences (Box 1) [11]. This suggests that failure to address pain early in the animal’s life not only compromises immediate welfare but could exacerbate distress during subsequent husbandry events such as shearing, handling, or transport later in life.

Box 1 **The Hidden Consequences of Untreated Pain.**

❖ Persistent sensitisation:
  - Pain left unmanaged can cause long-term changes in the nervous system, leading to heightened pain sensitivity during later procedures or injuries [3].
  - Repeated exposure to painful stimuli without analgesia promotes peripheral and central sensitisation, prolonging discomfort and recovery time [10].
❖ Early-life effects:
  - Painful experiences in neonatal lambs can alter nociceptive processing and behaviour into adulthood [10].
  - Ewes exposed to early-life pain (e.g., tail docking) showed more pain-related behaviours at parturition as adults, suggesting increase in pain sensitivity [12].
❖ Trans-generational consequences:
  - Offspring of ewes exposed to early-life pain or immune challenge displayed lower pain tolerance, implying potential epigenetic inheritance of pain sensitivity [12].

Though evidence remains limited, these findings indicate that painful experiences may influence welfare outcomes across generations

In extensive systems where re-handling is impractical, this under-recognition strengthens the case for analgesic strategies that align with both the biology of prolonged pain and the realities of on-farm management [13,14]. Cost and product availability also strongly influence producer decisions with veterinarians and farmers both reporting that drug prices, limited registered options and restricted access reduce willingness to implement pain management protocols [14,15]. In Australia, many systemic analgesics for sheep must be purchased through a veterinarian, in contrast to widely available over-the-counter products such as clostridial vaccines and the topical anaesthetic Tri-Solfen^®^ (Dechra Pharmaceuticals) which are more easily incorporated into routine husbandry practices. The oral trans-mucosal treatment of meloxicam (Butec^®^) is available over the counter and is increasingly used on-farm, highlighting how ease of access can influence analgesic uptake. In some cases, when approved products are unavailable, veterinarians must rely on extra-label drug use (use of a drug in a non-listed way on the label), creating added complexity around withdrawal periods and residue compliance [14,15]. Furthermore, research indicates that producers’ decisions to use analgesia are strongly influenced by their perception of its benefits. Several studies have reported only modest or inconsistent improvements in production outcomes, such as weight gain, following analgesic treatment [3]. Consequently, some producers perceive the cost of providing pain relief as unjustified. In contrast, others increasingly regard the use of analgesia essential for maintaining social license and meeting evolving animal welfare expectations [13].

Together, these findings highlight the barriers to analgesic adoption and the need for practical, cost-effective, and longer-lasting solutions that can be seamlessly integrated into routine husbandry practices.

## 3. Effective Analgesia: Defining What Works

In Australia, and indeed internationally, pain management options for sheep are restricted, with only a small number of approved analgesic products, predominantly Non- Steroidal Anti-Inflammatory Drugs (NSAIDs) and local anaesthetics, available for use.

The adoption of NSAIDs such as meloxicam has represented important progress in livestock welfare. Meloxicam, a preferential Cyclooxygenase 2 (COX-2) inhibitor, is widely used in cattle, pigs, and sheep, offering a favourable safety profile and clear evidence of analgesic benefit [10,16]. While meloxicam is an effective NSAID for on-farm use due to its demonstrated efficacy and relatively long plasma half-life compared with other drugs in its class, the duration of analgesia is shorter than the multi-day course of pain associated with husbandry procedures [4,10]. This mismatch between the physiological duration of pain and the pharmacological effect means that animals may experience continued discomfort once drug concentrations fall below effective levels [17]. Repeated dosing is ideal to maintain analgesic coverage, as prescribed for companion animals such as dogs after veterinary procedures [18] but it is rarely feasible on extensive farms where sheep are dispersed across large paddocks. Furthermore, repeated handling of sheep (mustering and restraint) in extensive systems increases stress and injury risk [19], labour, and cost which makes multi-dose options impractical on most commercial farms [3].

Another persistent limitation of meloxicam is that sheep-specific therapeutic levels remain undefined. Doses are often extrapolated from horses [20], despite the clear interspecies differences in COX inhibition potency and plasma concentration–time profiles [21]. A study in horses established an effective concentration range following an IV administration of meloxicam and identified 0.6 mg/kg as sufficient for clinical pain relief [20], but comparable data are lacking for sheep. Controlled studies in sheep show that 1.0 mg/kg meloxicam reduces pain-related behaviours [22], with no further benefit at higher doses. Buccal administration at the same dose also alleviated post-operative pain following castration and tail docking, though these behaviours re-emerged after 24–48 h, indicating that analgesia was shorter than the duration of pain [23]. It therefore remains uncertain whether current formulations maintain effective concentrations for the full duration of pain, a challenge that sustained-release meloxicam could address.

## 4. Sustained-Release Formulations: A Promising Direction

Unlike conventional formulations that provide brief exposure after a single dose, sustained-release (SR) formulations are designed to maintain therapeutic drug concentrations over a prolonged period [24]. These technologies were initially designed to reduce repeated dosing in companion and laboratory animals, but their relevance to production livestock, where handling and labour constraints are significant, has grown rapidly [25]. Several SR delivery strategies are currently under investigation for veterinary use such as polylactic-co-glycolic acid (PLGA) microspheres, which is a biodegradable polymer allowing controlled release as it gradually degrades [24].

Sustained release formulations of meloxicam have shown promising results in several species, providing extended analgesia with fewer administrations. In dogs, subcutaneous SR meloxicam maintained plasma concentrations above therapeutic thresholds for 48–72 h without injection-site reactions or systemic toxicity [26]. Similar success has been reported in pigs and horses, where oil-based or granulated sustained-release formulations of meloxicam prolonged absorption, maintained effective plasma levels for up to 48 h, and showed improved bioavailability and slower elimination compared with conventional products [27,28].

In sheep, a SR meloxicam formulation produced higher peak plasma concentrations and a slightly longer elimination half-life (~15 h) than conventional meloxicam after subcutaneous administration, yet plasma levels did not remain above the presumed therapeutic threshold of 400 ng/mL for the full 72 h [19]. A subsequent study evaluating a novel biodegradable SR formulation reported slower absorption and prolonged circulation, with lower peak plasma concentrations (1.58 µg/mL) and declining tissue levels after 52 h. The drug remained detectable until the end of the study (336 h), although it was uncertain whether therapeutic concentrations were maintained throughout [21]. While research in companion species shows that sustained-release NSAIDs can reduce injection frequency and maintain steady analgesia, there are limited ovine studies under field conditions [25]. Continued refinement of polymer composition, release kinetics, and bioavailability is required before SR meloxicam can be confidently adopted in commercial sheep production [21]. Nonetheless, the evidence to date supports its potential as a welfare-enhancing and management-efficient innovation that could bridge the gap between pharmacological efficacy and on-farm practicality.

However, notwithstanding these encouraging findings, the development and application of SR meloxicam formulations in food-producing species such as sheep present several important challenges that must be addressed before widespread adoption can be considered. Prolonged drug exposure raises questions regarding tissue residues and withdrawal periods, particularly where extended release from a subcutaneous depot may not be accurately reflected by plasma terminal half-life estimates derived from conventional formulations. In such cases, depot-driven absorption may result in detectable residues persisting beyond periods suggested by apparent elimination kinetics. Furthermore, while SR formulations may extend the duration of analgesia, they may not provide sufficiently rapid onset to manage acute procedural pain, potentially necessitating combination approaches that incorporate an immediate-release component alongside sustained delivery. These pharmacokinetic and formulation-specific considerations have direct implications for human food safety, underscoring the need for robust residue depletion studies and conservative withdrawal periods to ensure consumer protection. Addressing these challenges through careful formulation design and comprehensive pharmacokinetic and residue evaluation will be critical to realising the welfare and practical benefits suggested by current experimental data.

## 5. Conclusions

Sustained-release meloxicam represents a promising and welfare-relevant advance for pain management in sheep, not simply because it extends drug exposure, but because it aligns more closely with the biological duration of pain and the realities of extensive livestock systems. While current evidence supports its potential to provide longer-lasting analgesia, reduce handling stress, and enhance on-farm practicality, further optimisation of formulation type, reproducibility, and pharmacokinetic performance, as well as therapeutic validation under commercial conditions, is required before widespread adoption. In particular, research in sheep will be essential to establish species-specific therapeutic thresholds and ensure safe, reliable pain mitigation that is compatible with food-safety requirements. Importantly, uptake of sustained-release strategies will depend not only on pharmacological performance but also on demonstrated practicality, cost–benefit, and regulatory clarity. By reframing analgesia around how sheep experience pain and how they are managed in practice, sustained-release formulations offer a realistic pathway to narrowing the persistent gap between welfare expectations and on-farm implementation.

## Data Availability

No new data were created or analyzed in this study. Data sharing is not applicable to this article.

## Abbreviations

The following abbreviations are used in this manuscript:

COX 2	Cyclooxygenase 2
NSAID	Non-Steroidal Anti-inflammatory drugs
PLGA	polylactic-co-glycolic acid
